# The PARIGA Server for Real Time Filtering and Analysis of Reciprocal BLAST Results

**DOI:** 10.1371/journal.pone.0062224

**Published:** 2013-05-07

**Authors:** Massimiliano Orsini, Simone Carcangiu, Gianmauro Cuccuru, Paolo Uva, Anna Tramontano

**Affiliations:** 1 CRS4 Bioinformatics Laboratory, Science and Technology Park Polaris, Pula, Italy; 2 Department of Physics, La Sapienza University of Rome, Rome, Italy; University College Dublin, Ireland

## Abstract

BLAST-based similarity searches are commonly used in several applications involving both nucleotide and protein sequences. These applications span from simple tasks such as mapping sequences over a database to more complex procedures as clustering or annotation processes. When the amount of analysed data increases, manual inspection of BLAST results become a tedious procedure. Tools for parsing or filtering BLAST results for different purposes are then required. We describe here PARIGA (http://resources.bioinformatica.crs4.it/pariga/), a server that enables users to perform all-against-all BLAST searches on two sets of sequences selected by the user. Moreover, since it stores the two BLAST output in a *python-serialized-objects* database, results can be filtered according to several parameters in *real-time* fashion, without re-running the process and avoiding additional programming efforts. Results can be interrogated by the user using logical operations, for example to retrieve cases where two queries match same targets, or when sequences from the two datasets are reciprocal best hits, or when a query matches a target in multiple regions. The Pariga web server is designed to be a helpful tool for managing the results of sequence similarity searches. The design and implementation of the server renders all operations very fast and easy to use.

## Introduction

The BLAST program is at the base of a plethora of sequence similarity studies from genomics to proteomics. Ortholog-based function prediction [1], detection of the presence of similar domains in different proteins [2] and of common exons among alternatively spliced isoforms [3], [4], analysis of the presence of common regulatory signals in potentially functionally related proteins or genes [5], [6] are just some of most common problems that can be answered by carefully analysing the results of different database searches.

BLAST related tools are available both for downloading [7], [8] and by web applications [9], [10]. While the former is a more flexible scenario and allows users to perform searches against local customized databases, the latter usually just permits to query available databases (e.g. nr, uniprot, ESTdb, etc.), but generally provides a more structured result report. The first solution generally requires additional packages or *ad-hoc* solutions to manage and post-process BLAST results especially for long and complex queries. Several stand-alone tools are available (some examples are: Bioperl [11], Moulder [12], Zerg [13], Museqbox [14]). They run locally, offer both simple and complex ways of filtering results (mainly on the basis of parameters such as as *e-value, similarity,* etc.) and return parsed results as flat files or in a spreadsheet format. These packages generally have good performances in terms of speed and flexibility but require a certain level of computer knowledge. They have to be installed and often work in a command line fashion. Alternatively, some stand-alone tools as NOBLAST-JAMBLAST [15] and Batch Blast Extractor [16] provide a graphical interface to manage the results; they also run locally, are platform independent and do not require any computer knowledge. NOBLAST-JAMBLAST requires a MySQL server to be installed. This allows the user to sort and filter BLAST results and to perform queries on them via a graphical interface. An additional class of programs (BOV [17], PLAN [18], nuclearBLAST [19]) are available via web (or require a web server installation on Linux platform), let the users upload their own BLAST results as plain text files, and provide a user-friendly environment to filter and analyse the results. These applications generally work by storing BLAST results in a relational database, which, contrary to the case of NOBLAST-JAMBLAST [15] is not accessible to the user.

Available packages and web services are designed to simplify the use of database search tools for both programmatic and manual usage and all of them have specific features that make them appropriate for specific applications. However, so far, no tool is available for reciprocal blasting of two user defined sequences dataset and for an easy mining of results in an efficient and user friendly fashion. Yet, this is not an uncommon need in biology [20]–[25].

This led us to develop a powerful tool, named PARIGA (it comes from the Sardinian idiom: *“a pair of”*), that, given two user selected protein and/or nucleotide datasets, performs all-vs-all reciprocal BLAST searches [7] using each member of the first set as a query and the second set as the target database and vice versa and permits to quickly filter and analyze the results. It enables the user to interrogate and compare intra- and inter- BLAST results by performing logical operations on them. Moreover, the web implementation allows directly the parsing of BLAST results. The action of filtering is performed in real-time and it is completely reversible since it acts at level of results visualization; thus, it does not require additional computational steps and it does not provoke loss of data at each filtering phase.

## Methods

In our system, the BLAST outputs are stored in a *python-serialized-objects* relational database by a dedicated version of a python package named BlaSTorage [26]. In short, the system works by generating and manipulating a universal data structure, a JSON (JavaScript Object Notation) object, which is a lightweight data-interchange format, for each of the BLAST outputs generated with the Blast+ implementation [27]. The JSON object does not contain the alignments (the largest part of the outputs), while a dynamic generation of javascript code is used to keep the size of these objects rather small. Furthermore, searches, ranking and filtering operations can be performed on the client rather than on the server side thereby substantially improving the speed of response. In other words, the technical implementation of the system is such that the interactions between the user and the server are kept to a minimum. Although, in principle, the allowed size of the dataset is unlimited, in the current release we encourage an upper limit on the size of the two input files (an amount of 1000 proteins of about 300 residues each corresponds to about 100 MB of transferred JSONs), but it should be noted that this is only due to the limitations in the browser capability of displaying large amount of data (client-side), rather than to the computational load (server-side).

## Results and Discussion

**Figure 1:**
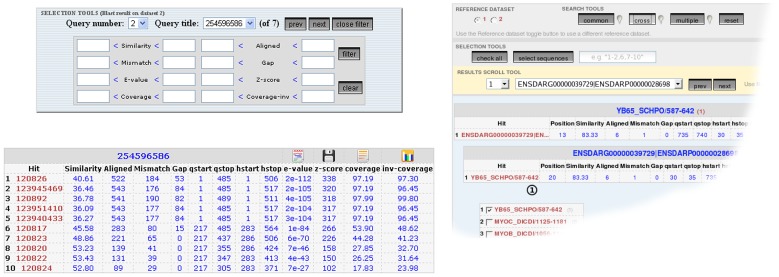
Real Time Filtering and Logical Operations. (Left) Real Time Filtering. Differently from other available tools, filtering of the returned results can be done in real time with PARIGA. By clicking the *filter* button, a form will appear where the user can insert the desired values (or ranges) and only filtered results will be shown. Four icons on the table header will show, from left to right, a graphical summary of the hits distribution on the query sequence, the results table, a Blast summary table and the Blast statistics. **(Right) Logical Operations.** The main result page will show three buttons that allow the user to perform logical operations between the two groups of results as described in the text.

**Figure 2:**
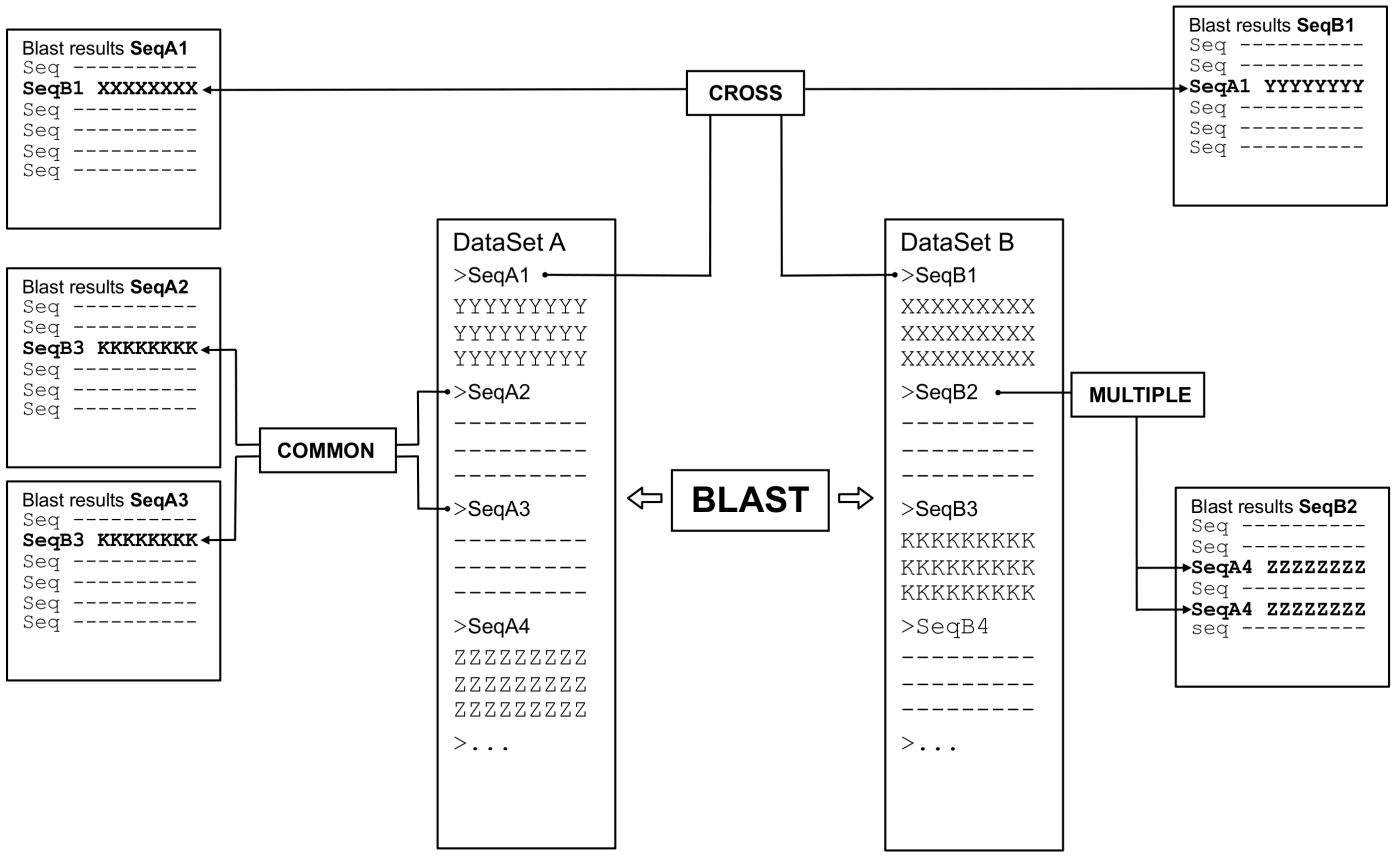
Pariga logical schema. Central columns represent the original input files, while results are indicated in the columns on the side. Boxes indicate logical operations that can be performed on the results. As an example: COMMON: which sequence(s) of the dataset B is(are) shared in BLAST results of sequence A2 and A3 of the dataset A? CROSS: once sequence A1 is selected from dataset A, in which results of the dataset B does it appear? MULTIPLE: which sequence of dataset A appears more than once (i.e. matches more than one region) in the results of sequence B2 of dataset B?

The two datasets can be uploaded by the user who can select the desired traditional BLAST parameters (*word size, expected e-value, gap-open and extension, etc.*). The visualization of each of the BLAST results is very similar to the standard tabular BLAST output with the possibility of retrieving the alignment by clicking on the entry name. Summary table, statistics and a graphical summary of matches can be viewed by clicking on the appropriate icon in the table header. Two additional columns have been added to the traditional schema, named *coverage* and *inv-cov*. These indicate the fraction of the query and the subject involved in the alignment, respectively. Two novel key functionalities have been implemented. First of all, the results can be visualized and filtered in real time according to parameters such as *similarity*, *coverage*, *e-value* etc. (Figure 1, left panel). Second, since results are stored in a hidden database structure, the user can easily perform logical operations on them by simply selecting one of the options COMMON, CROSS and MULTIPLE (Figure 1, right panel, and schema in Figure 2).

### Filtering Results

By clicking the *open filter* button a form will appear where the user can insert the upper and lower limits for the desired parameters (eg. similarity between 40% and 80%). This will cause a refresh of the results page where only the results satisfying the imposed set of parameters will be displayed. Also graphical results will be updated according to the filters. Multiple filtering criteria can be applied simultaneously. While both full raw and summarized Blast results can be downloaded by the link on the top of the page, the real time filtered table can be exported by clicking on the “Export Table” icon in the table header.

### COMMON Searches

The COMMON option allows the visualization of the hits of one dataset that are shared among the BLAST results of selected entries in the other dataset. The selection of the entries can be done via checkboxes or by typing the numbers corresponding to the indexes of the entries of interest. The system visualizes the results as a table where each hit is linked to the corresponding alignment, plus the BLAST parameters and the relative position of each hit in the query search.

The possible applications of this option include the possibility of identifying proteins sharing a similar domain or, conversely, to identify multiple domains present in the same set of proteins. It can also be used to ask which exons are present in which set of transcripts and their range of similarity, which could give insights into their mechanism of evolution. If a sequence sets is composed of putatively co-expressed or co-regulated genes and the other by regulatory sequences, the user can use this option to identify which sequences share the same regulatory sequences or, on the contrary, which regulatory sequences are present in which transcripts.

### CROSS Searches

The CROSS option allows the user to highlight and retrieve cases where two entries of the two input datasets are reciprocally matched as query-subject pair in the BLAST searches. One obvious application of this option is the detection of orthologous genes or proteins by allowing the quick identification of protein of gene pairs that represent the best hit for each other. Given the suggested upper limit in the number of input sequences, this search can be applied to the whole proteomes of small organisms (eg. bacteria), while for the eukaryote orthologue prediction a smaller set of sequences enriched in potential candidates has to be used.

### MULTIPLE Searches

Finally, the MULTIPLE option is used to verify whether one of the queries matches more than one region on the same entry of the second dataset. Also in this case, the potential applications are many and diverse, for example the detection of targets of the same microRNA, the identification of binding sites for multiple microRNAs on the same target gene or the detection of multiple protein domains in the same protein sequence. We also included a button to quickly filter all the queries which do not have multiple hits on the same subject, in order to guarantee a fluid navigation among results.

Detailed examples of the three options, including screenshots, are shown in Text S1. Several additional features complete the web application: it is possible to export the raw Blast results as text files, to perform a self-Blast simply leaving empty the second dataset (in this case the entries of the first dataset will be blasted against the dataset itself). Finally, the results of COMMON, CROSS and MULTIPLE searches, run across the entire dataset, are summarized in tables shown in pop-up windows.

### Ongoing Work

As we mentioned, even though theoretically this application is unlimited in terms of size of the datasets, we strongly suggest an upper limit of 5000 submitted sequences to ensure reasonable computational times since the visualization step strongly depends upon the user client. In our tests Pariga successfully run with 20,000 vs 20,000 blasts, although it required more than 60 minutes for the display of the results). This is most likely not a serious limitation since people dealing with larger dataset (e.g. at the genome scale) are unlikely to perform visual and interactive inspection of the BLAST results and prefer ad-hoc solutions to parse and manage Blast output according to the question. We developed Pariga with the aim of helping biologist dealing with small datasets, where Blast results have to be extensively explored. For larger datasets, command line solutions (i.e. based on database frameworks) could be more suitable. Moreover, for these tasks the Pariga engine can be applied [26]. We plan to develop the system further, by adding the possibility of performing TBLASTX and TBLASTN [8] searches and to extend its applicability to whole genome analysis by moving most of the computations to the server side.

### Conclusions

Similarity searches in protein and nucleotide sequence databases represent the starting point and often the foundation of many biological discoveries. We developed a tool to manage, query and compare results from reciprocal BLAST analysis.

### Availability

http://resources.bioinformatica.crs4.it/pariga/.

## Supporting Information

Text S1 **Pariga tutorial including two case studies with screenshots.**
(PDF)
