# Supplementary material for: The PARIGA Server for Real Time Filtering and Analysis of Reciprocal BLAST Results
Source: PLoS One. 2013 May 7;8(5):e62224. doi: 10.1371/journal.pone.0062224 (PMC3646873; doi:10.1371/journal.pone.0062224)
Supplement: Text S1 — Pariga tutorial including two case studies with screenshots. (PDF) [file pone.0062224.s001.pdf]

Pariga Tutorial

PARIGA TUTORIAL 1

BLAST Parameters 2

Input Data 2

Test Datasets 3

Run 3

Results 4

List of the sequences producing significant alignments, with score and E-value 5

Blast statistics for the current search 5

Logical operations 6

Common 7

Cross 7

Multiple 7

CASE STUDIES 7

Case study 1 – Identification of miRNA targets 7

Case study 2 – Identification of Pfam domains 14

REFERENCES 20

BLAST Parameters

The home page presents a basic interface that asks for the dataset type (Protein/Nucleic Acid) and automatically selects the corresponding BLAST program (blastp/blastn).

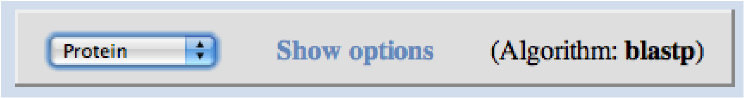

By clicking on "**Show Options**" several menus will appear where the user can modify the default values of the most common BLAST parameters. Clicking on "**Hide Options**" will restore the initial screen.

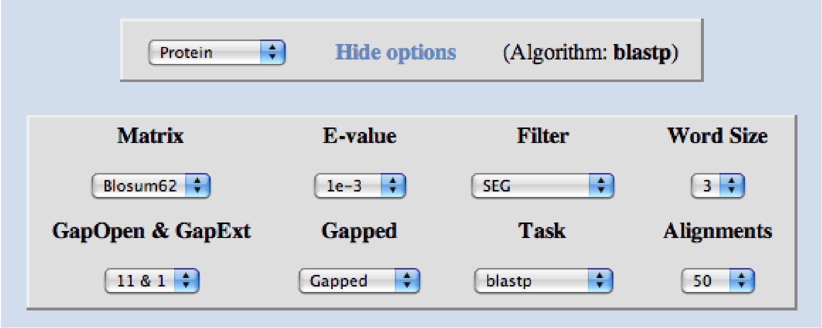

Input Data

According to the options selected in the previous section, the system will ask the user to upload a protein dataset or a DNA/RNA dataset.

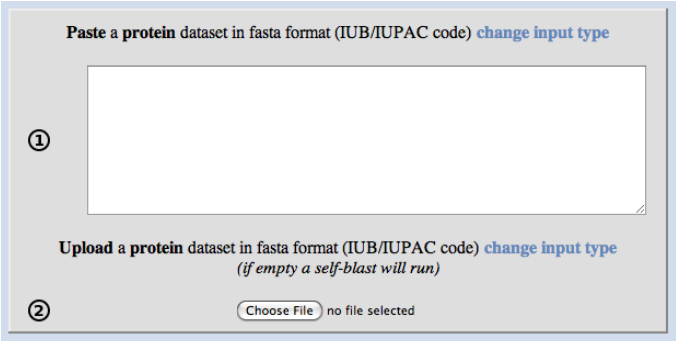

The two datasets can be uploaded as files or directly pasted in the input form. The two options are available by clicking the "**change input type**" button. If the second dataset is not provided, the software will perform a self-blast on the first dataset.

Test Datasets

Some example datasets are available in the "**test datasets**" section. Test datasets (nucleotide or amino acid sequences) can be selected via the scroll menu, and loaded into the input form by clicking on the "**Load**" button.

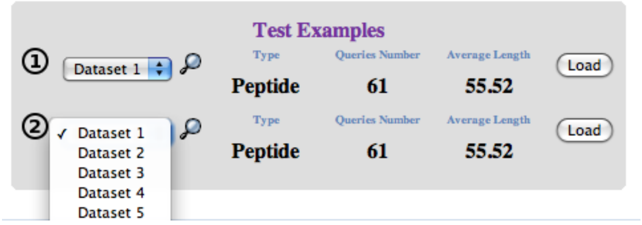

Run

The job is submitted by clicking the "**run**" button at the bottom of the page, and a new form with the **jobID** will appear. The **jobID** can be used to retrieve the results later.

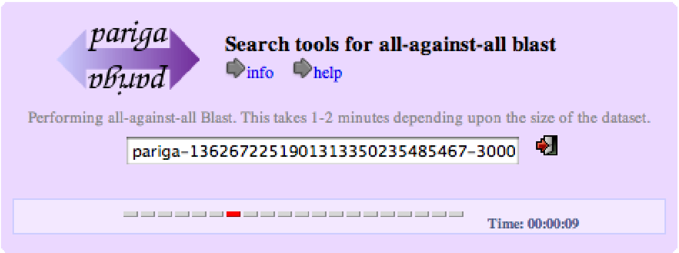

Results

When a job is completed, a page with two tables will appear with the results of the Blast searches of dataset 1 vs 2 and dataset 2 vs 1.

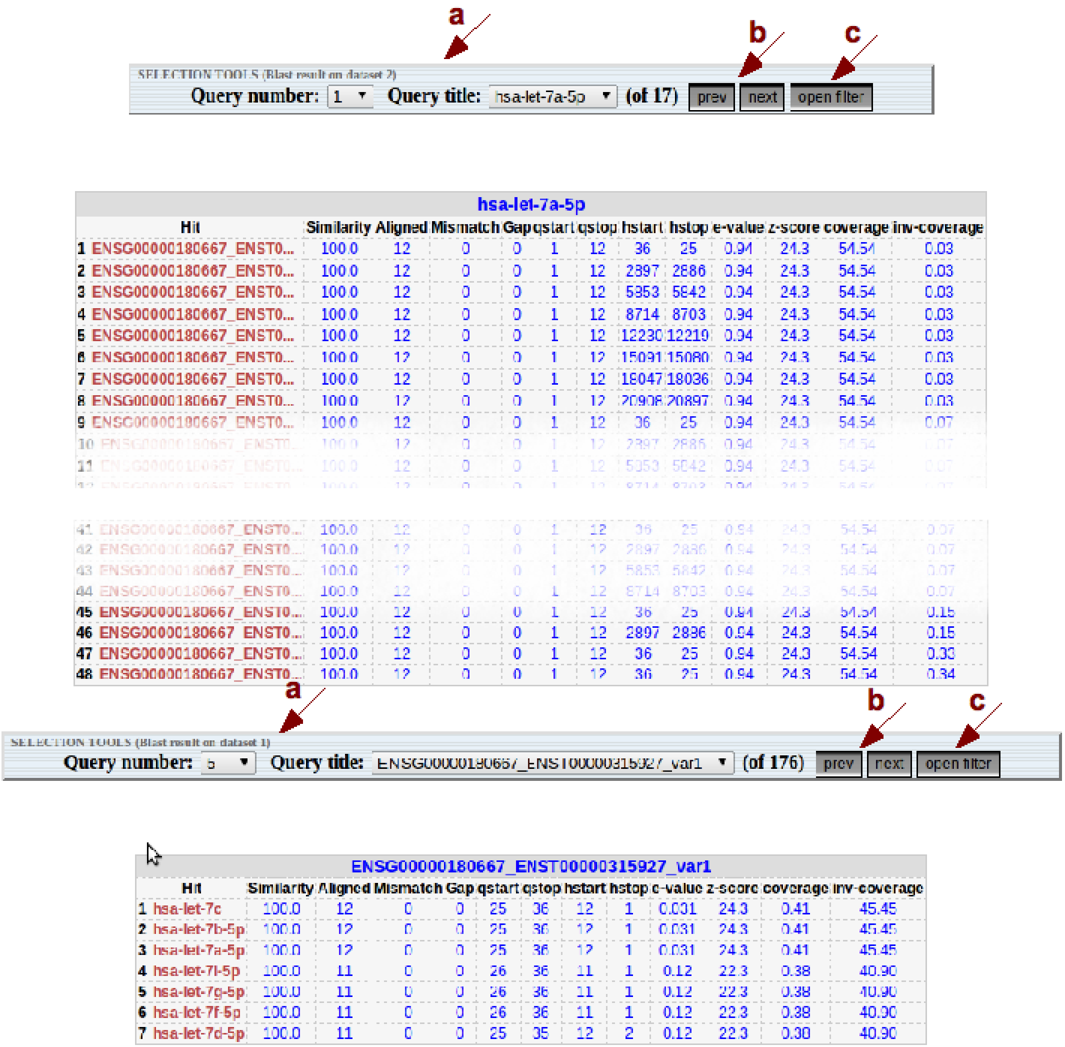

	hsa-let-7a-5p												
	Hit	Similarity	Aligned	Mismatch	Gap	qstart	qstop	hstart	hstop	e-value	z-score	coverage	inv-coverage
1	ENSG00000180667_ENST0...	100.0	12	0	0	1	12	36	25	0.94	24.3	54.54	0.03
2	ENSG00000180667_ENST0...	100.0	12	0	0	1	12	2897	2886	0.94	24.3	54.54	0.03
3	ENSG00000180667_ENST0...	100.0	12	0	0	1	12	5853	5842	0.94	24.3	54.54	0.03
4	ENSG00000180667_ENST0...	100.0	12	0	0	1	12	8714	8703	0.94	24.3	54.54	0.03
5	ENSG00000180667_ENST0...	100.0	12	0	0	1	12	12230	12219	0.94	24.3	54.54	0.03
6	ENSG00000180667_ENST0...	100.0	12	0	0	1	12	15091	15080	0.94	24.3	54.54	0.03
7	ENSG00000180667_ENST0...	100.0	12	0	0	1	12	18047	18036	0.94	24.3	54.54	0.03
8	ENSG00000180667_ENST0...	100.0	12	0	0	1	12	20908	20897	0.94	24.3	54.54	0.03
9	ENSG00000180667_ENST0...	100.0	12	0	0	1	12	36	25	0.94	24.3	54.54	0.07
10	ENSG00000180667_ENST0...	100.0	12	0	0	1	12	2897	2886	0.94	24.3	54.54	0.07
11	ENSG00000180667_ENST0...	100.0	12	0	0	1	12	5853	5842	0.94	24.3	54.54	0.07
[illegible]	[illegible]	[illegible]	[illegible]	[illegible]	[illegible]	[illegible]	[illegible]	8714	8703	0.94	[illegible]	[illegible]	[illegible]
41	ENSG00000180667_ENST0...	100.0	12	0	0	1	12	36	25	0.94	24.3	54.54	0.07
42	ENSG00000180667_ENST0...	100.0	12	0	0	1	12	2897	2886	0.94	24.3	54.54	0.07
43	ENSG00000180667_ENST0...	100.0	12	0	0	1	12	5853	5842	0.94	24.3	54.54	0.07
44	ENSG00000180667_ENST0...	100.0	12	0	0	1	12	8714	8703	0.94	24.3	54.54	0.07
45	ENSG00000180667_ENST0...	100.0	12	0	0	1	12	36	25	0.94	24.3	54.54	0.15
46	ENSG00000180667_ENST0...	100.0	12	0	0	1	12	2897	2886	0.94	24.3	54.54	0.15
47	ENSG00000180667_ENST0...	100.0	12	0	0	1	12	36	25	0.94	24.3	54.54	0.33
48	ENSG00000180667_ENST0...	100.0	12	0	0	1	12	36	25	0.94	24.3	54.54	0.34

	ENSG00000180667_ENST00000315927_var1												
	Hit	Similarity	Aligned	Mismatch	Gap	qstart	qstop	hstart	hstop	e-value	z-score	coverage	inv-coverage
1	hsa-let-7c	100.0	12	0	0	25	36	12	1	0.031	24.3	0.41	45.45
2	hsa-let-7b-5p	100.0	12	0	0	25	36	12	1	0.031	24.3	0.41	45.45
3	hsa-let-7a-5p	100.0	12	0	0	25	36	12	1	0.031	24.3	0.41	45.45
4	hsa-let-7i-5p	100.0	11	0	0	26	36	11	1	0.12	22.3	0.38	40.90
5	hsa-let-7g-5p	100.0	11	0	0	26	36	11	1	0.12	22.3	0.38	40.90
6	hsa-let-7f-5p	100.0	11	0	0	26	36	11	1	0.12	22.3	0.38	40.90
7	hsa-let-7d-5p	100.0	11	0	0	25	35	12	2	0.12	22.3	0.30	40.90

The header of each table contains two menus (**Query number** and **Query title**) to jump to a particular result ("a"). Alternatively the "**prev-next**" buttons allow the user to scroll over the results ("b"). The "**open filter**" button ("c") opens a form where the user can select the minimum, maximum or both values for the desired parameters. By clicking the "**filter**" button, only matches satisfying the filters will be displayed.

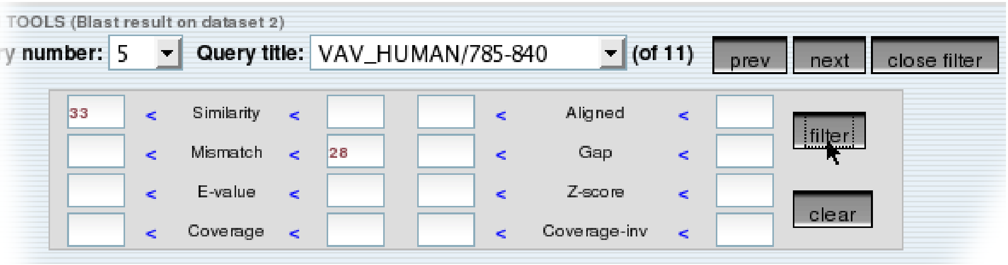

The sequence alignment of the individual hits can be visualized by clicking on the hit name.

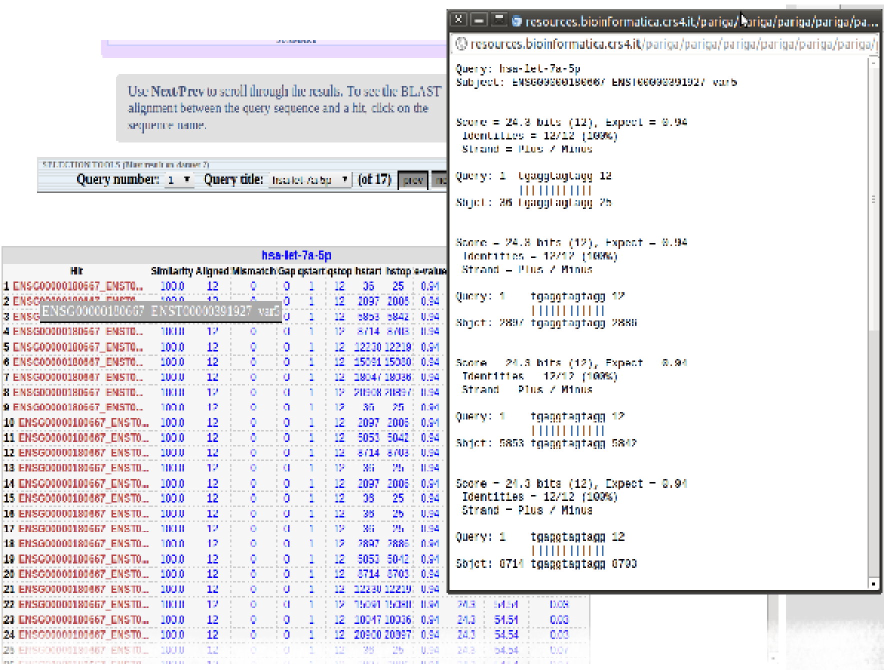

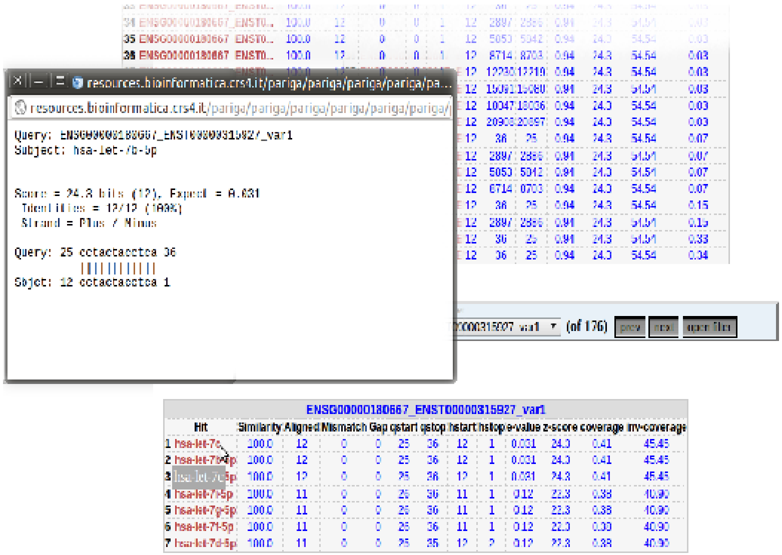

Selecting one the four icons available at the top of each table, a pop-up window with additional information will be displayed:

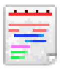 Graphical report of the distribution of the Blast hits on the query sequence, where hits are colored according to the alignment score.

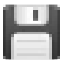 Export of the current table.

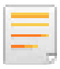 List of the sequences producing significant alignments, with score and E-value.

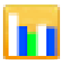 Blast statistics for the current search.

Logical operations

Pariga is able to perform logical operations with the results of the two Blast searches. The tools can be accessed by clicking the "**search tools**" link at the top of the result page.

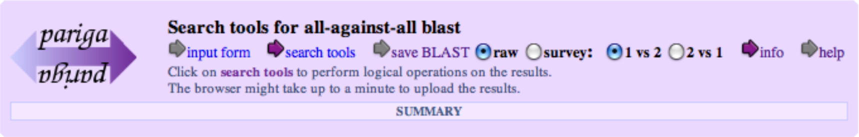

This section enables the user to apply three different search criteria:

- **Common**: Select two or more sequences in the first data set to find out whether they match the same sequence(s) in the second dataset
- **Cross**: Select one sequence in the first data set to find if there is a reciprocal match with sequence(s) in the second dataset
- **Multiple**: Select one sequence in the first data set to find out whether they match more than one region in the same sequence(s) in the second data set

An overall summary of the results obtained with the different queries is available in the **Summary Results** section: the **Common** summary table contains a list of sequences with common hits; the **Cross** summary table contains the results of a cross search for each sequence; the **Multiple** summary table contains a list of sequences with multiple hits on the same sequence.

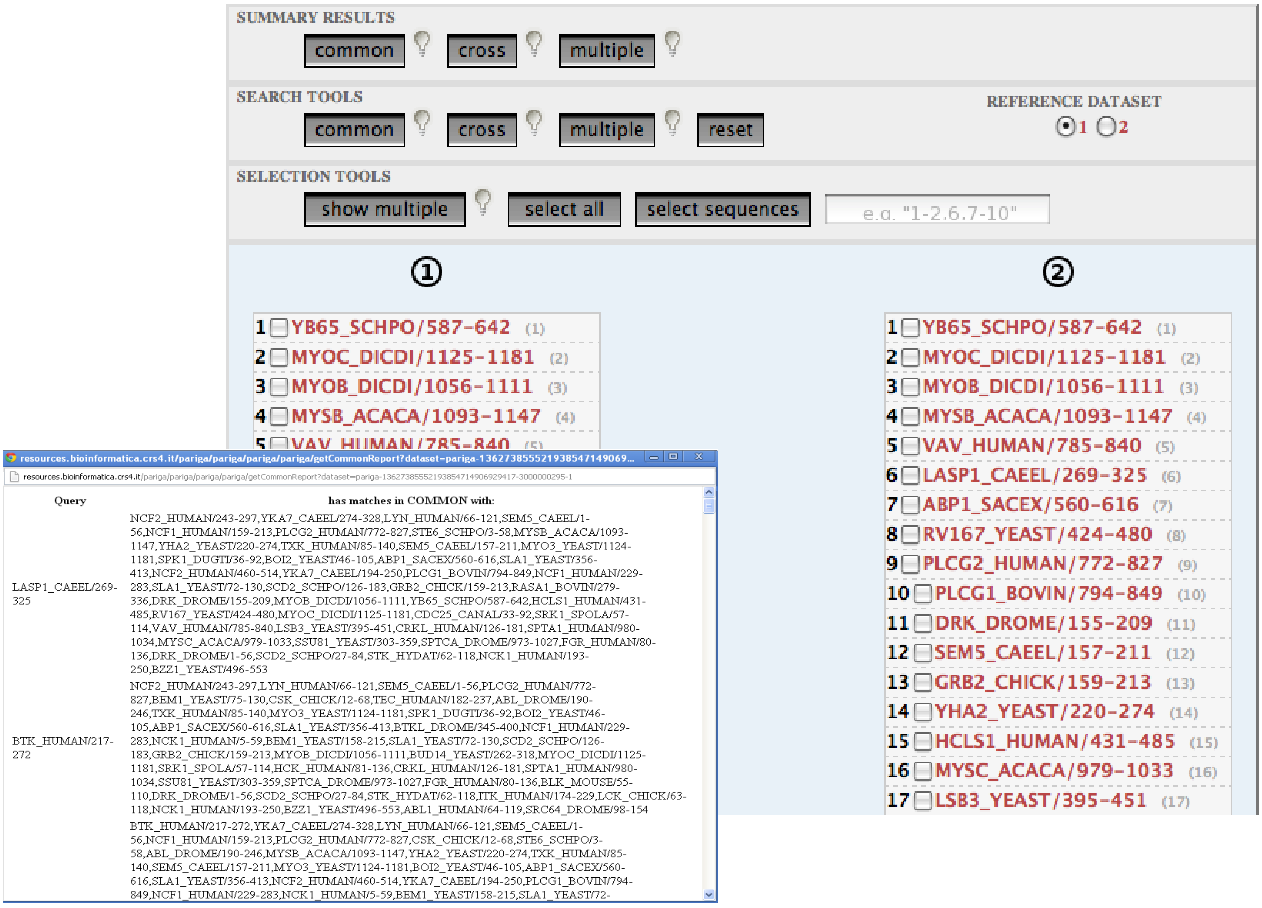

By using the options available in the **Selection Tools** section it is possible to quickly select/unselect a set of sequences or filter for sequences that match more than one region on the same entry (**show multiple**).

The reference dataset for the summary results and the individual searches can be selected using the **Reference Dataset** buttons. To perform a new search and discard the results of the previous one, the **reset** button has to be clicked. A contextual help ( ) is associated to each button.
The two-columns table contains the names of the sequences in the two datasets that can be selected for the different searches.

Common

The **Common** search checks whether two (or more) sequences in the reference dataset (identified by the checkbox in the table header, "**reference dataset**") share common results among the matched sequence in the other dataset. By indicating the number corresponding to the sequences or selecting them via the checkboxes and clicking the "**common**" button, a table with results will appear.
This table will indicate the name of the common matched sequence in the header, and the result parameters for each of the selected sequences. If more than one sequence in the dataset satisfies the logical query, the "**next-prev**" buttons can be used to scroll through the results.
The "**open filter**" button allows the user to filter the results as previously described. The alignment will appear in a pop-up window by clicking on the sequence name.

Cross

The **Cross** search allows checking whether the selected sequence in the reference dataset is reciprocally matched by a sequence in the other dataset. Sequence selection is carried out as described in the previous section. Clicking the "**cross**" button will display two tables showing the reciprocal blast results. The "**prev-next**" pairs of buttons will allow scrolling over the results. The alignment will appear in a pop-up window by clicking on the sequence name.

Multiple

The **Multiple** search will check if the selected sequence in the reference dataset matches sequence(s) in the other dataset in more than one region. Once selected the reference dataset and the desired sequence, clicking the "**multiple**" button will show a table with the results, if any. The "**prev-next**" pairs of button will allow scrolling over the results. The alignment will appear as pop-up window by clicking on the sequence name.

CASE STUDIES

Case study 1 – Identification of miRNA targets

Recent studies have shown that, in some forms of tumors, several genes elude the miRNA-based repression by a mechanism based on the alternative splicing of the polyadenylation signal [1]. In practice, two (or more) transcript splicing isoforms differ for the length of their 3'UTR only, one with the "canonical" length, the other(s) with a shortened variant downstream a secondary polyadenilation signal. While the former contains multiple sites for miRNA pairing the latter includes just one (or fewer) miRNA pairing site(s). The second isoform can then elude the miRNA driven repression.

We decided to investigate if targets of the miRNA family let-7 (predicted by TarBase [2]) contain multiple potential miRNA pairing sites across different polyadenylation sites in their 3' sequences. We show here how we took advantage of PARIGA to investigate the problem.

Dataset 1: mature sequences of miRNA let-7 family, 17 sequences (downloaded from miRBase [3])

Dataset 2: 3'UTR sequences of transcripts of top 20 predicted target genes (according to TarBase), downloaded through Ensembl web sites [4] and processed using an *ad-hoc* python script to generate all possible variants terminating the original sequence at every polyadenylation signal (AAUAAA as indicated by [5]). This resulted in176 variants.

BLAST parameters: default, expected value < 0.01

Results

As shown in the header of the first table, only 9 miRNAs had a match. The first is the hsa-let-7a-5p. Due to the table dimensions the full name of the matched sequences is truncated but it can be shown by passing over the names with the mouse.

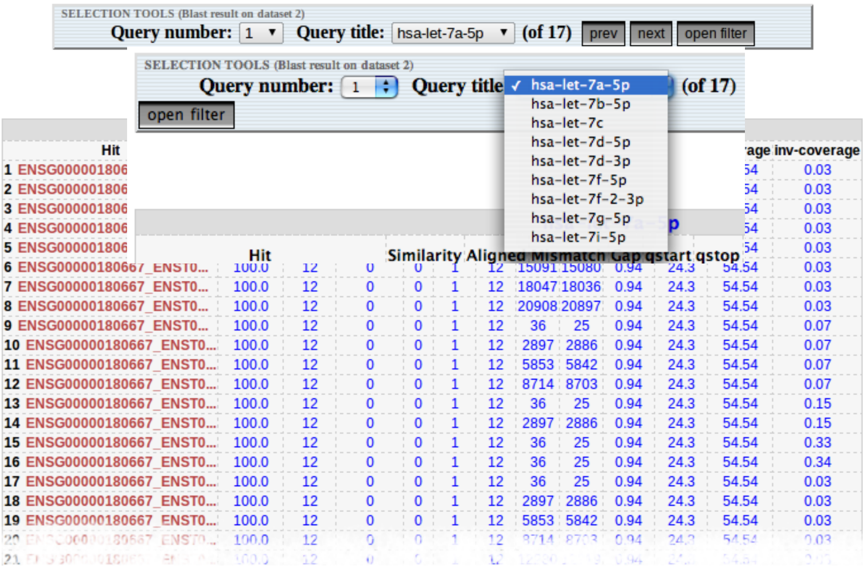

All matches had 100% identity and 12 aligned nucleotides, but different variants were matched in different regions. For example, the first 8 hits belong to the ENST00000391927 variant number 5 (the columns *hstart-hstop* indicates the coordinates), results 9-12 instead belong to variant number 4, results 13-14 to variant number 3, results 15 to variant number 2, result 16 to variant number 1, etc.

By performing the reciprocal analysis, only 37 sequences out of 176 had a match in the dataset 1. For example, in the first result, the ENST00000315927_var1, had a perfect match (100%) with 7 miRNAs, three of them with an aligned region of 12 nucleotides and the others with 11 aligned nucleotides. All of them are good candidates for mRNA repression since the perfectly aligned region maps the mirna's 5' [3] as can be noted in the *hstart-hstop* columns.

The results can be scrolled by clicking the "**prev-next**" button.

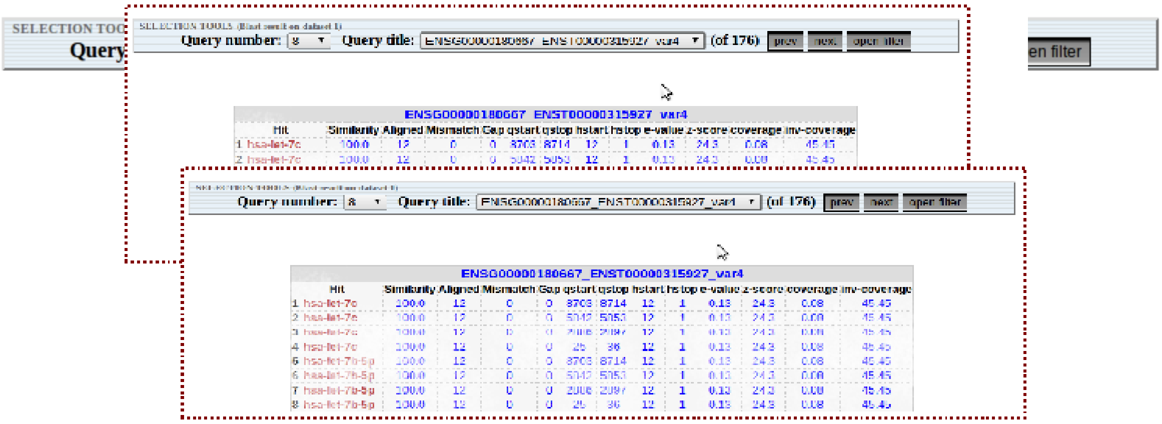

As described before, alignments can be retrieved by clicking on the sequence names.

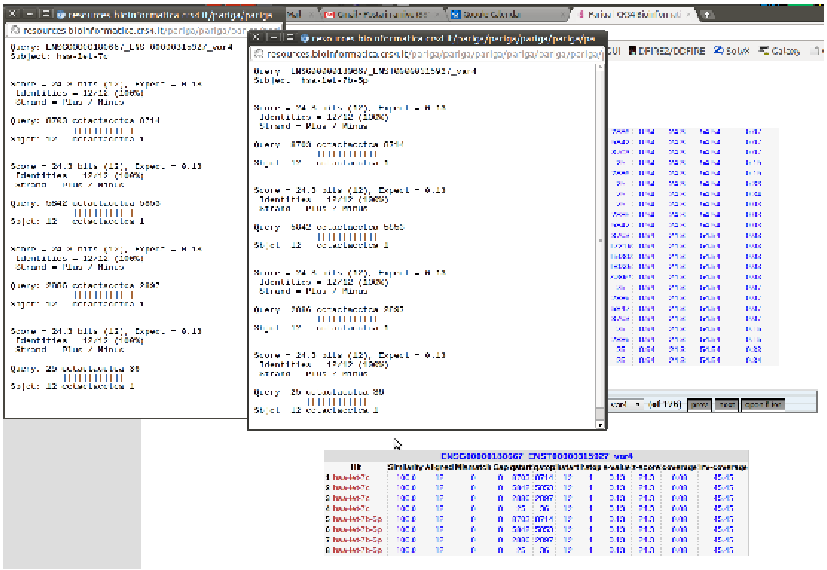

Results can be further explored using the "**search tools**".

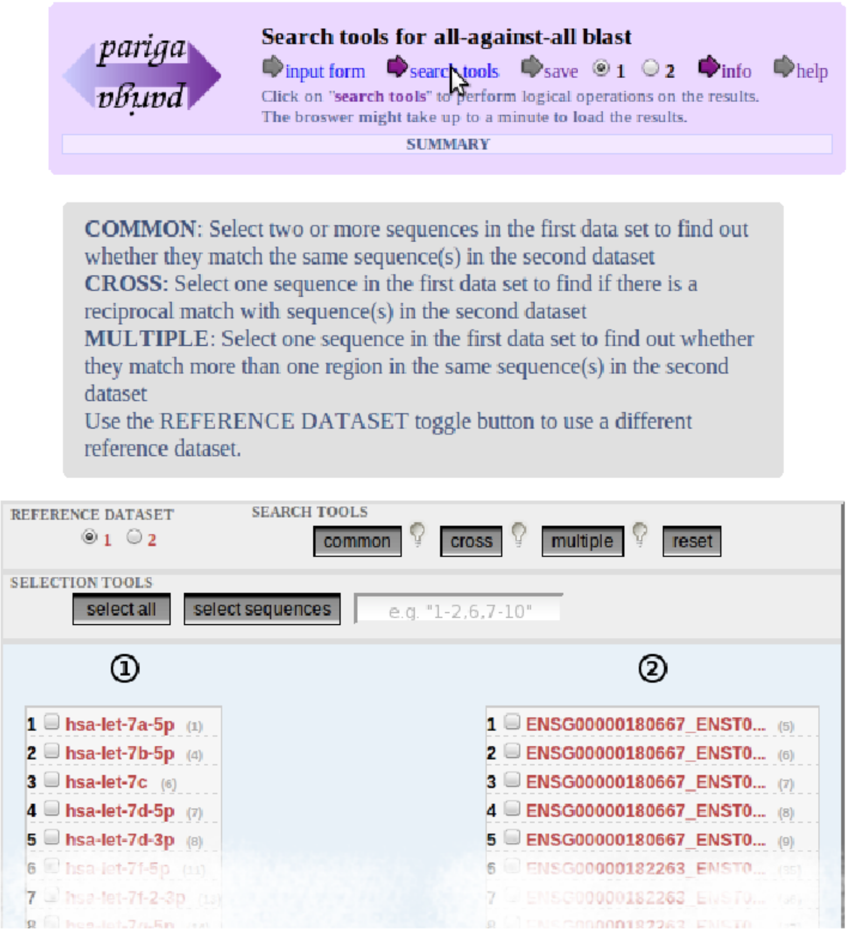

For example, we can investigate whether *hsa-let-7a-5p* and *hsa-let-7b-5p* share a common target. First, we select the reference database, then the two miRNAs by clicking on the checkboxes near their names. By clicking the "**common**" button a table appears showing that the sequence ENST00000315927_var1 is matched by the two selected miRNAs at the same potential binding site (positions 25-36, columns *hstart, hstop* in the table).

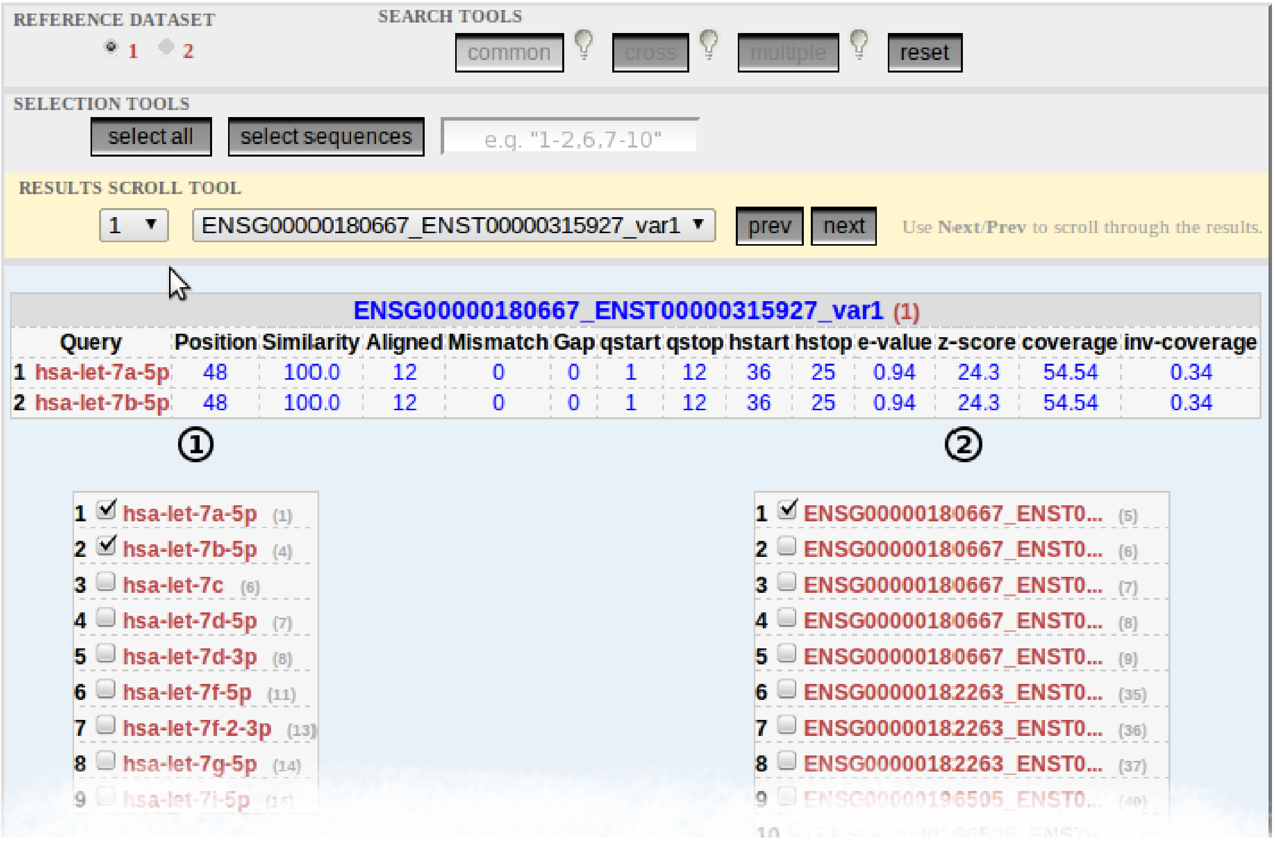

Using the “”**prev-next**” buttons we can browse the other results:

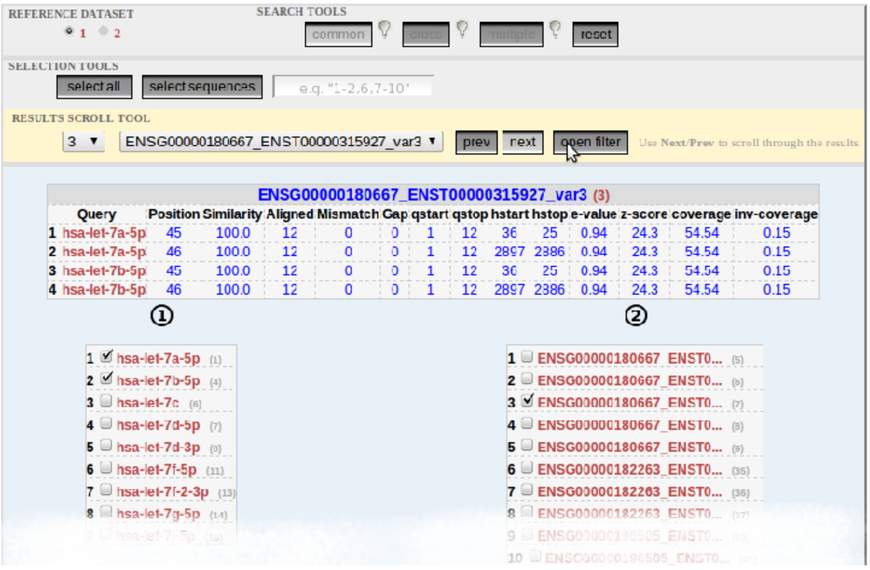

We could also investigate, for a given mirna (es *hsa-let-7c*), which sequence is reciprocally blasted. First of all we have to select the reference database by selecting 1 in the **Reference Dataset** section, and the miRNAs by clicking on the checkboxes near their names. Then, by clicking the “**cross**” button, two tables will appear showing the results for the two blast searches, and highlighting only the two involved sequence. As an example in the pair considered here, the blast search of the *hsa-let-7c* against the 3’ dataset shows that the ENST00000315927_var5 has 8 matching regions of twelve nucleotides each (*qstart,qstop* columns in the first table) and viceversa. The ranking in the relative blast searches is indicated in the column “*position*” while the numbers in the parenthesis in the headers of the tables indicate the position of that sequence in the original dataset.

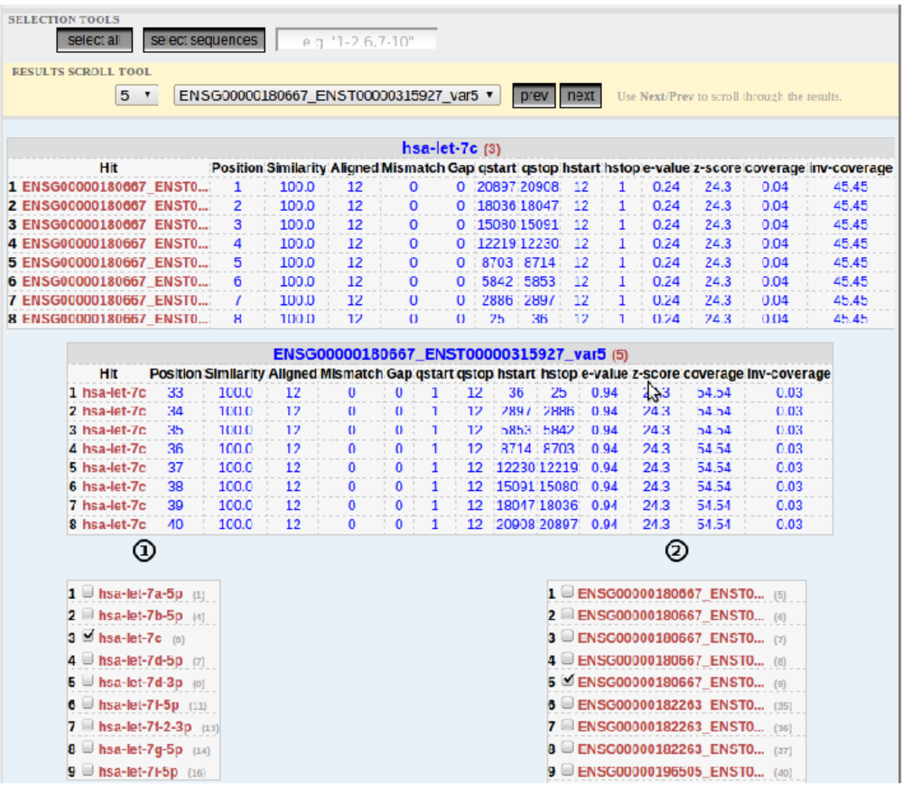

The results of a similar search for the mirna *hsa-let-7d-5p* is shown in the following figure, where the scroll menu to navigate among results is highlighted.

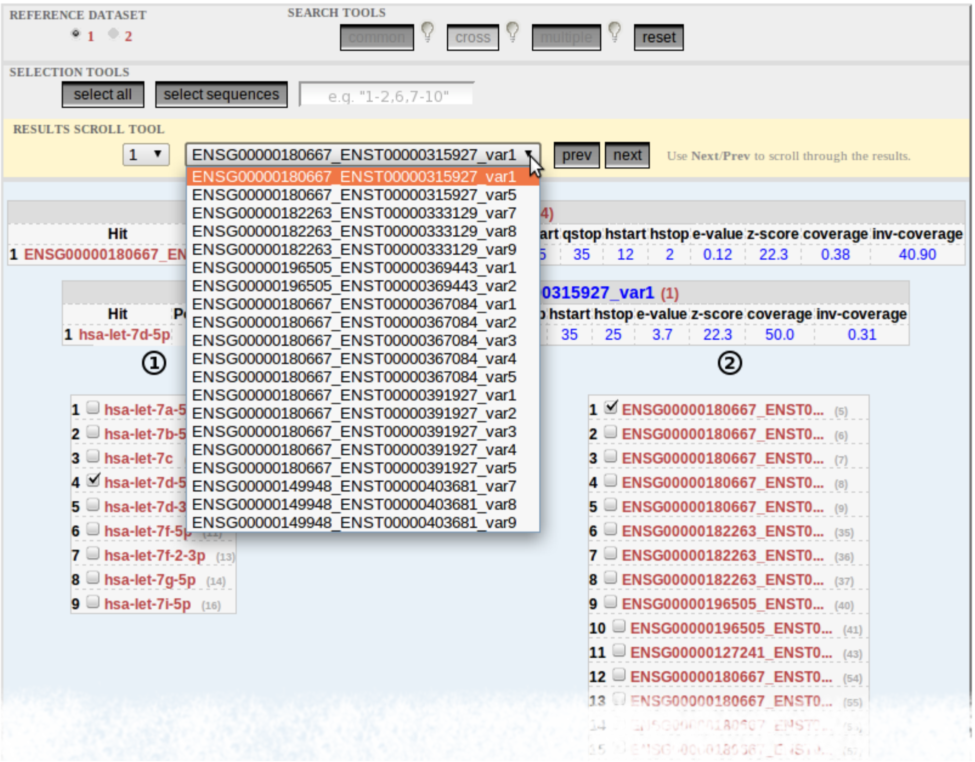

Finally, a further application could be the identification of UTR sequences which are matched by a given miRNA (the *hsa-let-7d-5p* in the figure) more than once. As usual, we have to select the reference database, then the desired miRNA, followed by clicking on "**multiple**" button. In this example, nine UTR sequences are matched by the selected miRNA more than once: in the

following figure, ENST00000315927_var3 is matched by the *hsa-let-7d-5p* miRNA at the positions 25-36 and 2886-2897 (columns *hstart,hstop*). The scroll menu can be used to select other results.

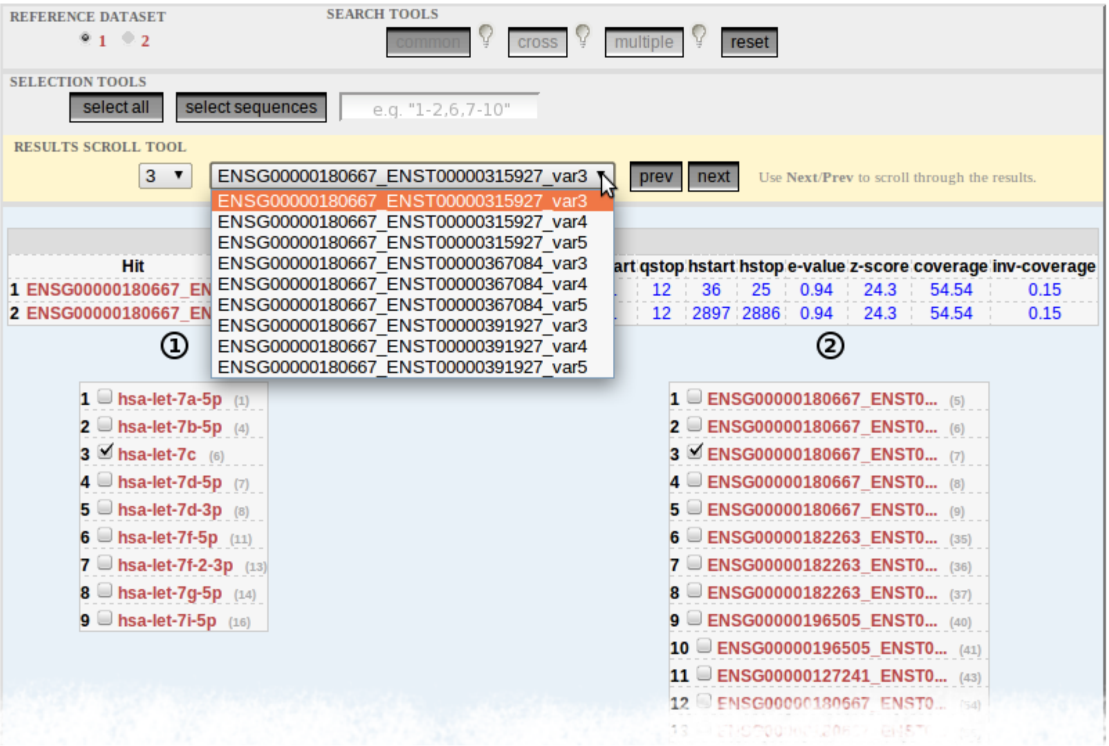

Case study 2 – Identification of Pfam domains

Let us assume that we have several protein sequences for which no functional experimental evidences is known, or that are derived from in silico studies. We can investigate whether any of these peptides shows sequence similarity with annotated DNA topoisomerase I protein sequences.

We can compare these sequences with seed sequences of the Pfam Topisom_I_N domain (PF02919), the DNA binding fragment of eukaryotes DNA topoisomerase I [6].

Dataset 1: putative “novel proteins” obtained by Ensembl [4] and filtered for length > 200 aa. Result: 454 sequences

Dataset 2: seed sequences (48) of PFAM domain PF02919 from the PFAM web site

BLAST parameters: default, expected value < 0.1

Results

After running, the system will display:

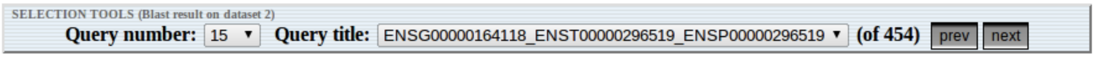

	ENSG00000164118_ENST00000296519_ENSP00000296519											
Hit	Similarity	Aligned	Mismatch	Gap	qstart	qstop	hstart	hstop	e-value	z-score	coverage	inv-coverage
1 Q8I3Z9_PLAF7_154-380	30.0	80	37	19	117	193	125	188	0.021	26.9	19.74	28.19

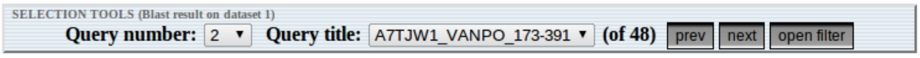

	A7TJW1_VANPO_173-391											
Hit	Similarity	Aligned	Mismatch	Gap	qstart	qstop	hstart	hstop	e-value	z-score	coverage	inv-coverage
1 ENSG00000259455_ENST0...	33.89	59	38	1	94	152	484	541	0.022	30.4	26.94	6.79
2 ENSG00000184206_ENST0...	36.17	47	29	1	94	139	221	267	0.050	29.3	21.00	8.18
3 ENSG00000215749_ENST0...	34.04	47	30	1	94	139	270	316	0.084	28.5	21.00	8.76
4 ENSG00000174450_ENST0...	45.45	33	18	0	94	126	308	340	0.084	28.5	15.06	3.63
5 ENSG00000259243_ENST0...	34.04	47	30	1	94	139	214	260	0.084	28.5	21.00	8.54
6 ENSG00000215749_ENST0...	34.04	47	30	1	94	139	256	302	0.084	28.5	21.00	9.00
7 ENSG00000174450_ENST0...	45.45	33	18	0	94	126	35	67	0.084	28.5	15.06	10.50
8 ENSG00000174450_ENST0...	45.45	33	18	0	94	126	308	340	0.084	28.5	15.06	5.07

We can see that the protein ENSP00000296519 has a single match against the Q8I3Z9 protein (topoisomerase I from *Plasmodium falciparum*), where 80 residues are aligned with a similarity of 30% and 37 mismatches.

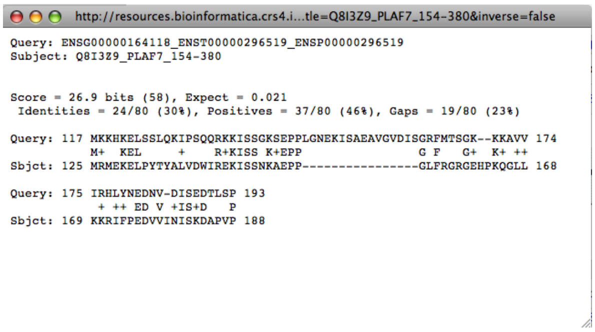
http://resources.bioinformatica.crs4.i...tle=Q8I3Z9_PLAF7_154-380&inverse=false

```
Query: ENSG00000164118_ENST00000296519_ENSP00000296519
Subject: Q8I3Z9_PLAF7_154-380


Score = 26.9 bits (58), Expect = 0.021
 Identities = 24/80 (30%), Positives = 37/80 (46%), Gaps = 19/80 (23%)

Query: 117 MKKHKELSSLQKIPSQQRKKISSGKSEPPLGNEKISAEAVGVDISGRFMTSGK--KKAVV 174
           M+  KEL     +    R+KISS K+EPP                G F   G+  K+ ++
Sbjct: 125 MRMEKELPYTYALVDWIREKISSNKAEPP----------------GLFRGRGEHPKQGLL 168

Query: 175 IRHLYNEDNV-DISEDTLSP 193
            + ++ ED V +IS+D   P
Sbjct: 169 KKRIFPEDVVINISKDAPVP 188
```

Other results can be displayed using the "**prev-next**" button or by directly selecting the query name in the scroll menu.

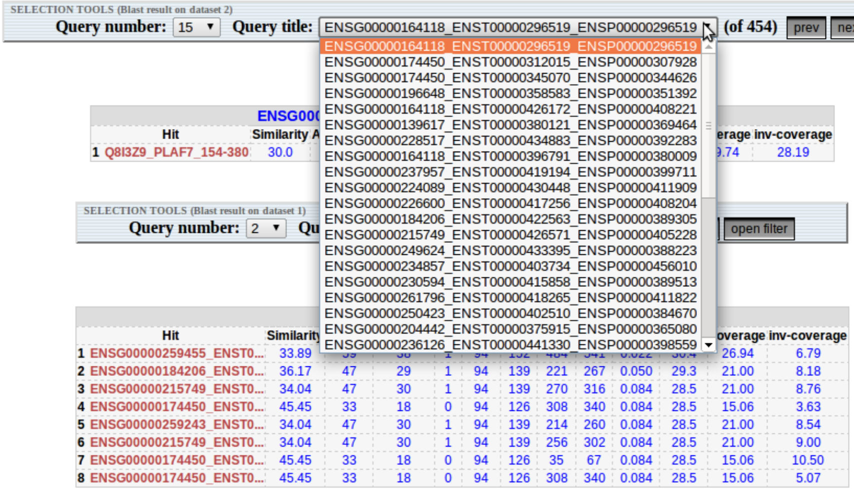

Sequences having no match do not appear in the menus.

By clicking the "**search tool**" button as previously indicated the two-column page will appear. As it can be observed seen, only 13 of the 48 domain sequences submitted appear in results.

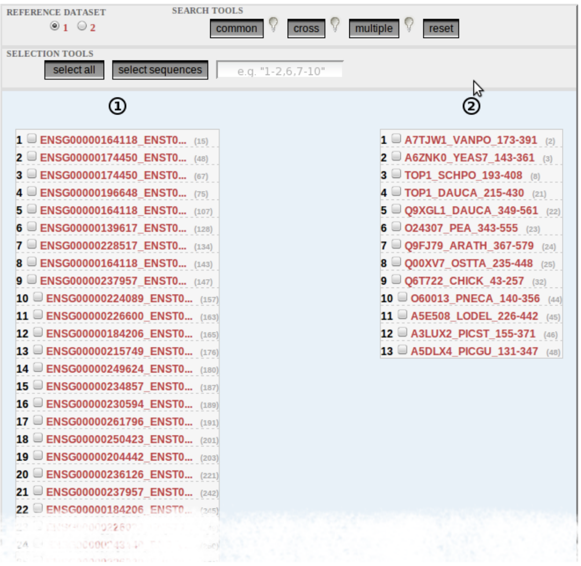

In this case there are no common results between the first and second search and therefore the "**common**" option returns the following message:

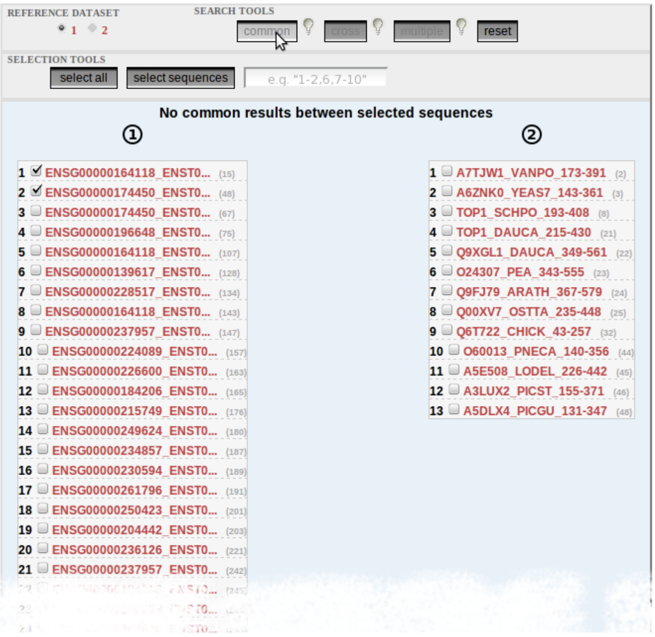

On the contrary, the two splicing variant ENSP00000307928 and ENSP00000454207 share common results. It is noteworthy that both proteins belong to the gene ENSG00000174450, which has four splicing variants in the dataset.

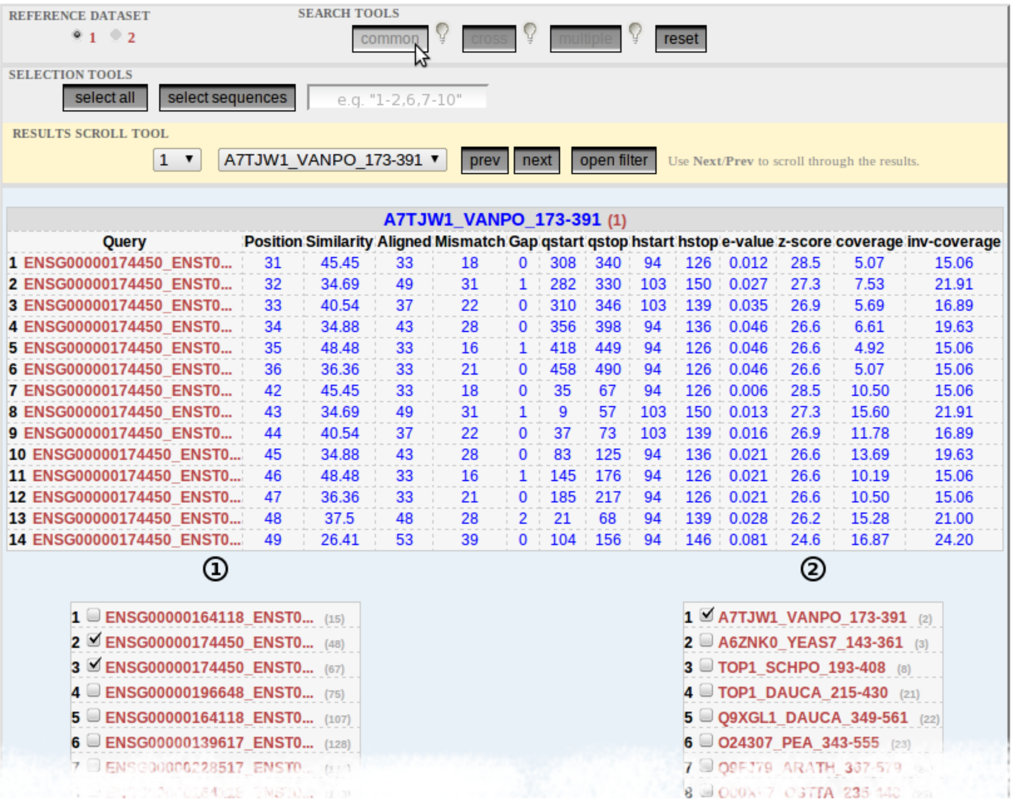

A7TJW1_VANPO_173-391 (1)

	Query	Position	Similarity	Aligned	Mismatch	Gap	qstart	qstop	hstart	hstop	e-value	z-score	coverage	inv-coverage
1	ENSG00000174450_ENST0...	31	45.45	33	18	0	308	340	94	126	0.012	28.5	5.07	15.06
2	ENSG00000174450_ENST0...	32	34.69	49	31	1	282	330	103	150	0.027	27.3	7.53	21.91
3	ENSG00000174450_ENST0...	33	40.54	37	22	0	310	346	103	139	0.035	26.9	5.69	16.89
4	ENSG00000174450_ENST0...	34	34.88	43	28	0	356	398	94	136	0.046	26.6	6.61	19.63
5	ENSG00000174450_ENST0...	35	48.48	33	16	1	418	449	94	126	0.046	26.6	4.92	15.06
6	ENSG00000174450_ENST0...	36	36.36	33	21	0	458	490	94	126	0.046	26.6	5.07	15.06
7	ENSG00000174450_ENST0...	42	45.45	33	18	0	35	67	94	126	0.006	28.5	10.50	15.06
8	ENSG00000174450_ENST0...	43	34.69	49	31	1	9	57	103	150	0.013	27.3	15.60	21.91
9	ENSG00000174450_ENST0...	44	40.54	37	22	0	37	73	103	139	0.016	26.9	11.78	16.89
10	ENSG00000174450_ENST0...	45	34.88	43	28	0	83	125	94	136	0.021	26.6	13.69	19.63
11	ENSG00000174450_ENST0...	46	48.48	33	16	1	145	176	94	126	0.021	26.6	10.19	15.06
12	ENSG00000174450_ENST0...	47	36.36	33	21	0	185	217	94	126	0.021	26.6	10.50	15.06
13	ENSG00000174450_ENST0...	48	37.5	48	28	2	21	68	94	139	0.028	26.2	15.28	21.00
14	ENSG00000174450_ENST0...	49	26.41	53	39	0	104	156	94	146	0.081	24.6	16.87	24.20

Further, these two peptides have multiple matches against different sequences in the PF02919 seed dataset. As usual they can be showed by the scroll menu. Limits of the regions are listed in the *qstart-qstop* columns.

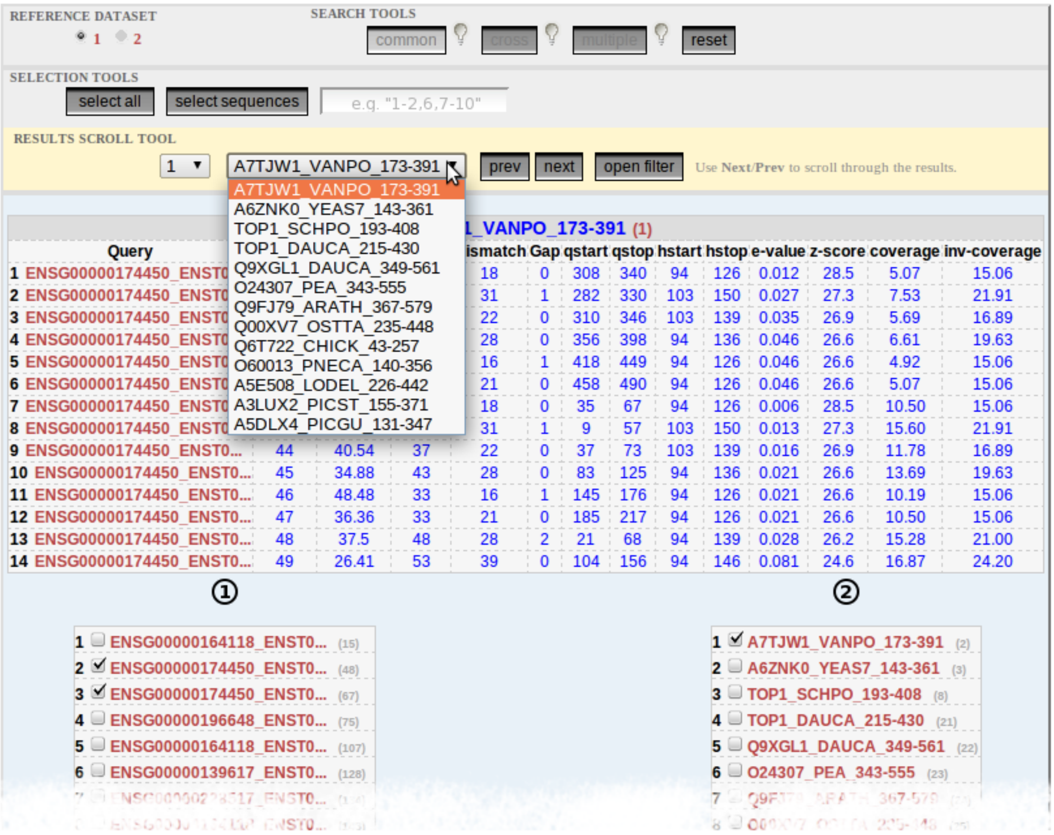

If we would like to know whether, for a given sequence, there are reciprocal hit matches we can select the sequence and then click the "**cross**" button.

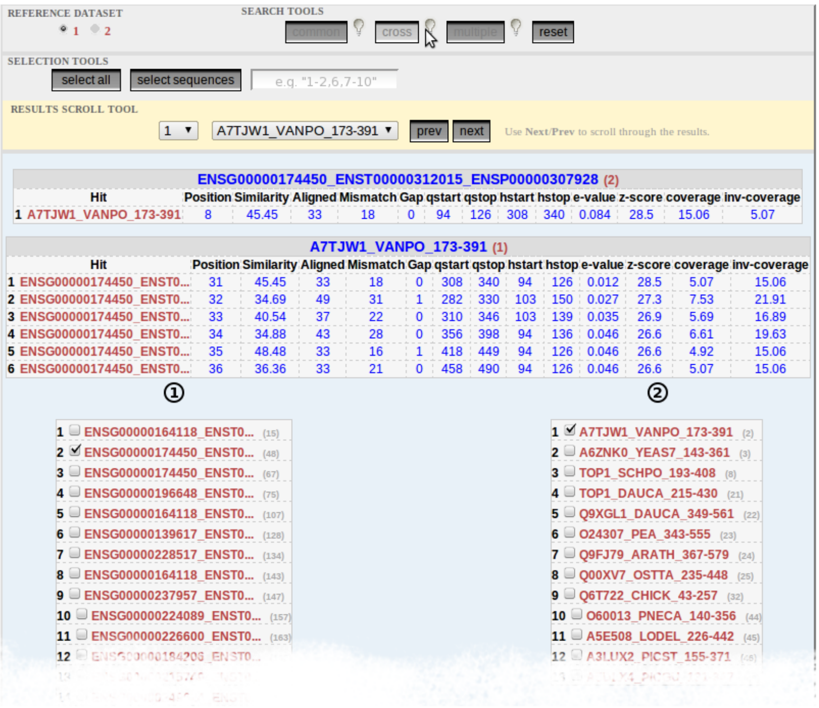

In the case shown in the figure, the two reciprocal hit sequences are the ENSP00000307928 protein and the A7TJW1 putative topoisomerase from *Vanderwaltozyma polyspora*.

It can be noted that while the protein matches the domain sequence just once (Table 1), the domain sequence matches the protein in different regions (Table 2) with different similarity and length of the alignment.

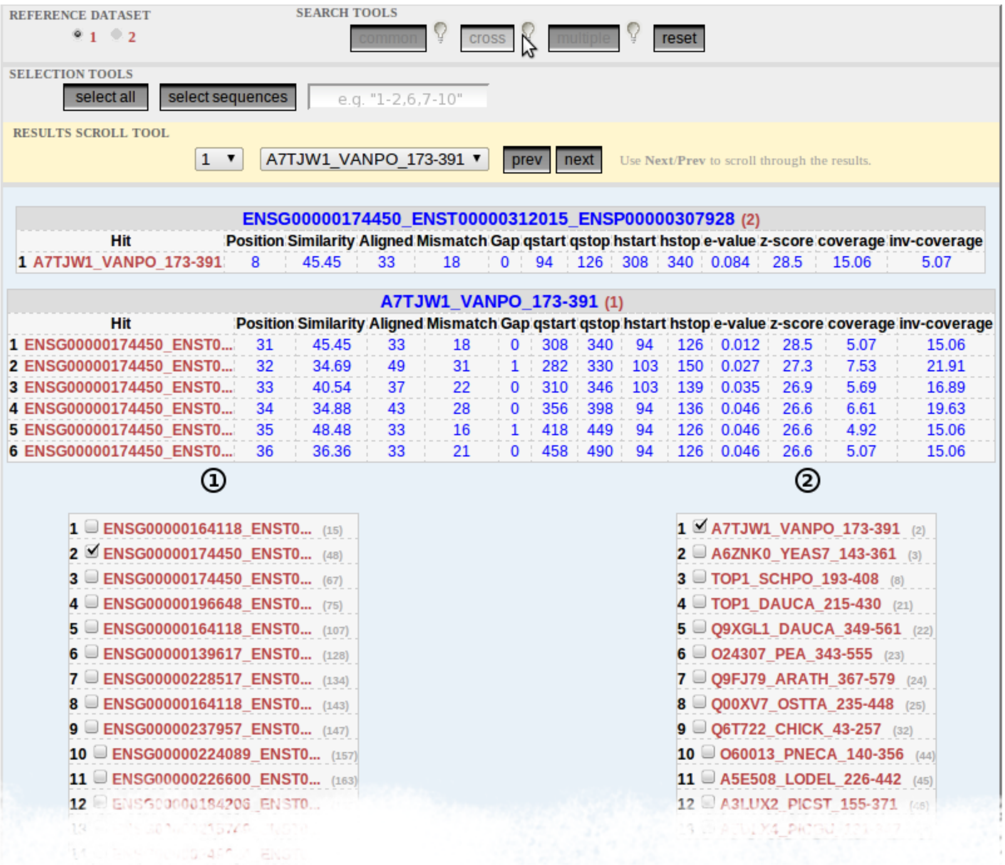

Also in this case, when no sequences are cross matched a message will appear.

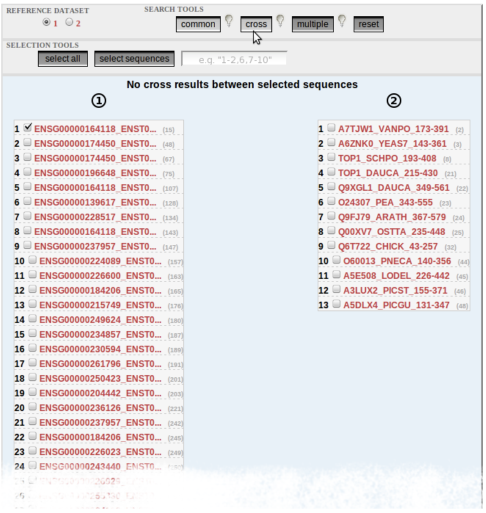

As we noted before the ENSP00000307928 protein is matched by the A7TJW1 sequence domain more than once. If we would like to further investigate this aspect we can use the "**multiple**" option. First, we click on the sequence name and then on the "**multiple**" button: a table with the regions matched by the query sequence will be shown in the result table.

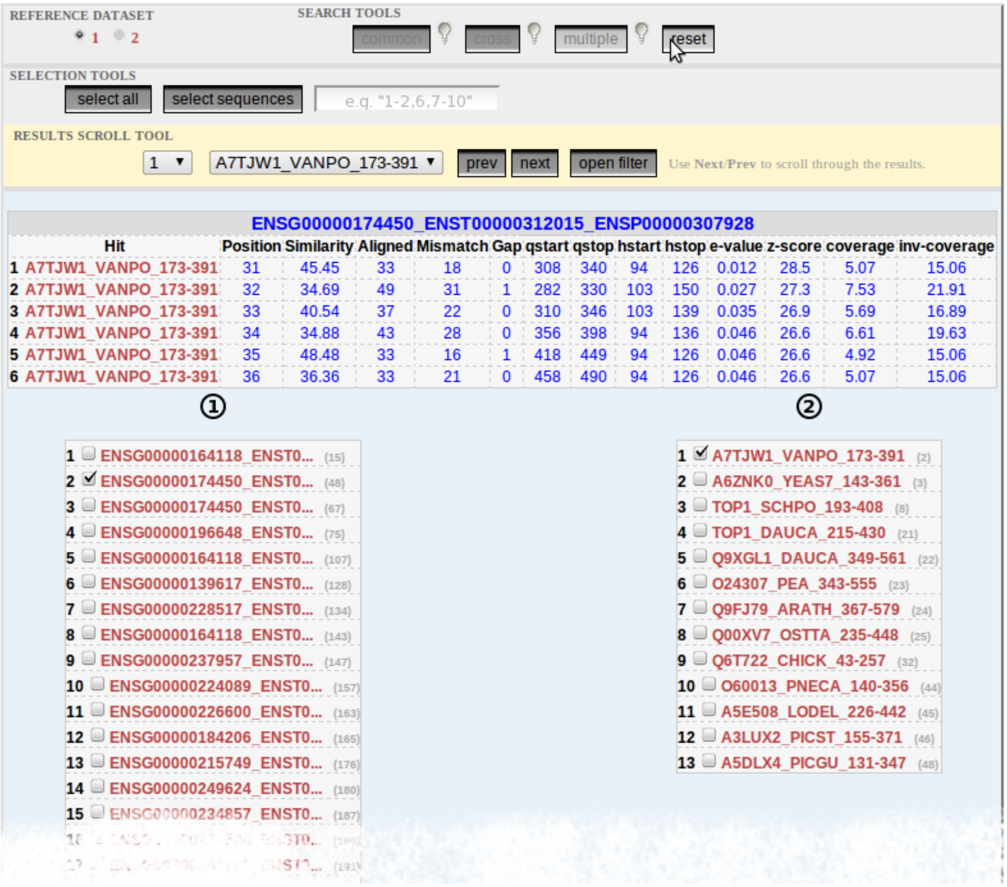

As mentioned before, these two sequences have multiple matching regions. The column *position* indicated where these results rank in the blast searches, while the columns *qstart,qstart* show where the alignments occur. Also in this case, if no multiple sequences occur, a message will be displayed.

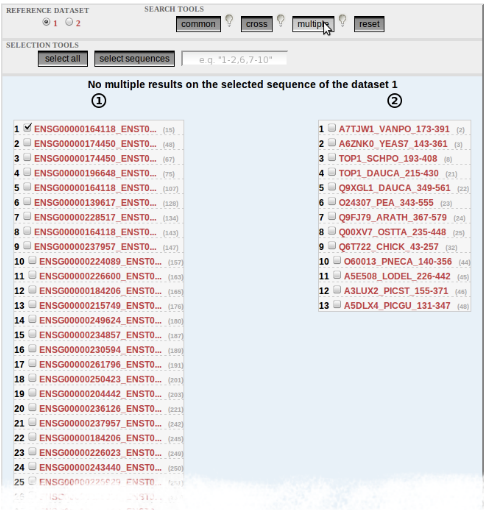

References

1. Sandberg R, Neilson JR, Sarma A, Sharp PA, Burge CB. Proliferating cells express mRNAs with shortened 3' untranslated regions and fewer microRNA target sites. Science 320: 1643-1647
2. Tarbase: [http://Diana.cslab.ece.ntua.gr/tarbase/]
3. Mirbase: [http://www.mirbase.org]
4. Ensembl: [http://www.ensembl.org]
5. Edmonds M. A history of poly A sequences: from formation to factors to function. Prog Nucleic Acid Res Mol Biol 2002, 71: 285-389.
6. Pfam: Eukaryotic DNA Topoisomerase I [http://www.pfam.sanger.ac.uk/family/pf02919]
